# Biological and Clinical Aspects of an Olive Oil-Based Lipid Emulsion—A Review

**DOI:** 10.3390/nu10060776

**Published:** 2018-06-15

**Authors:** Wei Cai, Phillip C. Calder, Maria F. Cury-Boaventura, Elisabeth De Waele, Julie Jakubowski, Gary Zaloga

**Affiliations:** 1 Shanghai Key Laboratory of Pediatric Gastroenterology and Nutrition, Shanghai Institute for Pediatric Research, Shanghai 200092, China; caiw1978@163.com; 2 Human Development and Health Academic Unit, Faculty of Medicine, University of Southampton, Tremona Road, Southampton SO16 6YD, UK; P.C.Calder@soton.ac.uk; 3 NIHR Southampton Biomedical Research Centre, University Hospital Southampton NHS Foundation Trust and University of Southampton, Tremona Road, Southampton SO16 6YD, UK; 4 Interdisciplinary Post-Graduate Program in Health Sciences, Cruzeiro do Sul University, Rua Galvão Bueno, 868, Sao Paulo 01506-000, Brazil; maria.boaventura@cruzeirodosul.edu.br; 5 Department of Intensive Care Medicine and Department of Nutrition, UZ Brussel, Vrije Universiteit Brussel (VUB), 1090 Brussels, Belgium; Elisabeth.DeWaele@uzbrussel.be; 6 TA Integrated Pharmacy Solutions, Baxter International Inc., One Baxter Parkway, DF5-3E Deerfield, IL 60015, USA; 7 Consultant Medical Affairs, Baxter Healthcare Corporation, One Baxter Parkway, Deerfield, IL 60015, USA; gpzaloga@aol.com

**Keywords:** parenteral nutrition, olive oil, oxidative stress, lipid peroxidation, lipid emulsions, immune system, hepatobiliary function

## Abstract

Intravenous lipid emulsions (ILEs) have been an integral component of parenteral nutrition for more than 50 years. Numerous formulations are available and are based on vegetable (soybean, olive, coconut) and animal (fish) oils. Therefore, each of these formulations has a unique fatty acid composition that offers both benefits and limitations. As clinical experience and our understanding of the effects of fatty acids on various physiological processes has grown, there is evidence to suggest that some ILEs may have benefits compared with others. Current evidence suggests that olive oil-based ILE may preserve immune, hepatobiliary, and endothelial cell function, and may reduce lipid peroxidation and plasma lipid levels. There is good evidence from a large randomized controlled study to support a benefit of olive oil-based ILE over soybean oil-based ILE on reducing infections in critically ill patients. At present there is limited evidence to demonstrate a benefit of olive oil-based ILE over other ILEs on glucose metabolism, and few data exist to demonstrate a benefit on clinical outcomes such as hospital or intensive care unit stay, duration of mechanical ventilation, or mortality. We review the current research and clinical evidence supporting the potential positive biological and clinical aspects of olive oil-based ILE and conclude that olive oil-based ILE is well tolerated and provides effective nutritional support to various PN-requiring patient populations. Olive oil-based ILE appears to support the innate immune system, is associated with fewer infections, induces less lipid peroxidation, and is not associated with increased hepatobiliary or lipid disturbances. These data would suggest that olive oil-based ILE is a valuable option in various PN-requiring patient populations.

## 1. Introduction

Parenteral nutrition (PN) is recognized as an important means to provide nutrition for patients who are unable to receive sufficient oral or enteral nutrition [1]. Parenteral nutrition should include a balance between glucose, amino acids, and lipids, as well as vitamins, minerals, and trace elements, in order to meet individual nutritional needs of patients. Lipids are an integral component of PN as they are rich in calories and provide essential fatty acids [1]. Several different formulations of intravenous lipid emulsions (ILEs) are commercially available: soybean oil-based ILEs (e.g., Intralipid^®^); mixtures of soybean long-chain triglycerides (LCT)/coconut oil medium-chain triglycerides (MCT) (MCT/LCT; e.g., Lipofundin^®^); olive oil-based ILE (olive oil 80%, soybean oil 20%, e.g., ClinOleic^®^); and fish oil-based ILEs either as a 100% ILE (e.g., Omegaven^®^) that is usually mixed with another ILE at the point of care, or preformulated fish oil-based ILEs such as Smoflipid^®^ (soybean oil 30%, MCT 30%, olive oil 25%, fish oil 15%) or Lipoplus/Lipidem^®^ (soybean oil 40%, MCT 50%, fish oil 10%).

Olive oil-based ILE has been commercially available since the 1990s and is widely used in some settings (see Table 1 for summary). The primary fatty acid in olive oil-based ILE is the omega (n)-9 monounsaturated fatty acid (MUFA), oleic acid. MUFAs have been associated with health benefits [2,3], and diets rich in MUFAs have been associated with a lower risk of inflammatory disease [4]. Oleic acid contributes approximately 60% of the total fatty acids in the 80% olive oil-based ILE [5]. Olive oil-based ILE also includes linoleic acid, an essential omega (n)-6 polyunsaturated fatty acid (PUFA), which contributes approximately 20% of the total fatty acids in the olive oil-based ILE [5]. Olive oil-based ILE also includes the essential omega (n)-3 PUFA α-linolenic acid, which contributes approximately 2% of the total fatty acid content [5]. Concerns have emerged regarding excess n-6 PUFAs and their effects on lipid peroxidation, immune function, and inflammation [6,7,8,9,10]. 

This narrative review summarizes the evidence for the effects of olive oil-based ILE on immune function and lipid peroxidation in vitro, in vivo (animal and human), and in clinical studies. Additionally, the effects of olive oil-based ILE on plasma lipids and glucose metabolism, hepatobiliary and endothelial function, and morbidity and mortality in clinical trials are summarized.

## 2. Literature Search

### 2.1. Literature Search Strategy

The Medline and Embase databases (inception to 15 September 2017) were searched using the terms (parenteral nutrition or PN) AND olive AND (lipid* OR oil* OR emulsion* OR ILE OR IVLE). The search was limited to English-language articles. Animal, in vitro, and in vivo studies, as well as prospective clinical studies (randomized and observational) in adult and pediatric patients, were included. Conference abstracts, case studies, and editorials were excluded. Review articles retrieved during the literature search were hand searched to identify any further articles of relevance.

### 2.2. Literature Search Results

**Figure 1:**
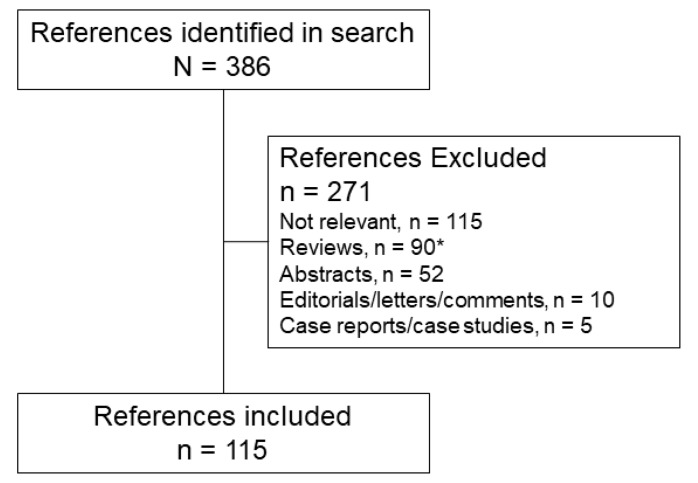
Flow diagram of the articles identified and included in this review. * Bibliographies of review articles were searched by hand to identify additional relevant articles.

A total of 387 articles were retrieved, of which 115 were included in the review (Figure 1). Most excluded articles were deemed irrelevant, primarily because they did not discuss olive oil-based ILE.

## 3. Immune Function

**Figure 2:**
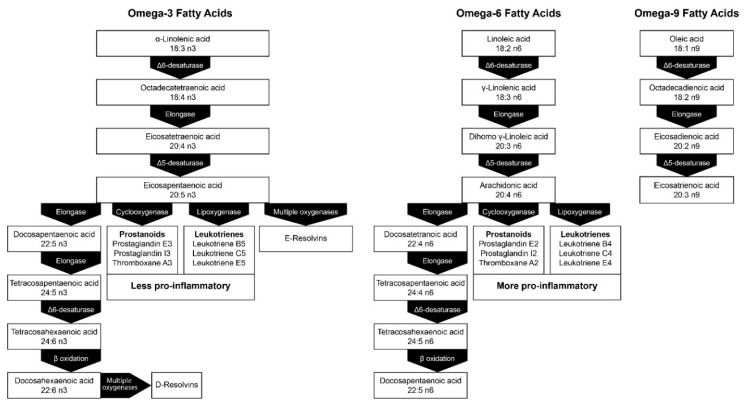
Metabolic pathways of *n*-3 and *n*-6 fatty acids. Adapted from [30], Copyright 2009, with permission from Elsevier.

Parenteral nutrition is commonly administered to critically ill patients who have inflammation and/or immune dysfunction and are therefore at an increased risk of infection. Fatty acid content of the ILE used for PN may impact immune function, and therefore the risk of infection and of organ damage arising from inflammation. Metabolism of n-6 PUFAs and n-3 PUFAs contributes to the formation of inflammatory mediators such as prostaglandins, leukotrienes, and resolvins (Figure 2), and some of these inflammatory mediators may play a role in increasing inflammation. Lipid emulsions containing high levels of n-6 PUFAs, such as soybean oil, have been reported to suppress the immune system [7], increase inflammation [8,9,10], and may be associated with higher rates of infection [20] compared with other ILEs. In contrast, lipid emulsions containing olive oil, which is high in the n-9 MUFA oleic acid, may preserve immune function [21]. 

### 3.1. Immune Response

Collective evidence from animal studies, in vitro cultured immune cell studies, and clinical studies suggests that olive oil-based ILE appears to preserve immune function (Table 2). Some studies have reported that olive oil-based ILE has no effect or beneficial effects on immune cell proliferation and function and/or immune cell death [7,22,23,24,25,26,27], as well as lesser effects on disruption of bacterial clearing [28,29] compared with other ILEs. Numerous studies have reported that oleic acid has direct effects on both the innate and adaptive immune systems (see review by Carillo et al., 2012) [4]. These include effects on the expression of cellular adhesion molecules, neutrophil aggregation, neutrophil phagocytic activity, neutrophil reactive oxygen species (ROS) production, leukocyte migration, T-cell proliferation, and lymphocyte apoptosis [4].

Analysis of the effects of olive oil-based ILE on isolated human lymphocytes and neutrophils revealed that olive oil-based ILE decreased lymphocyte proliferation, induced lymphocyte necrosis, and did not alter the proportion of viable neutrophils [22]. When these results were compared with a previous study by the same group using soybean oil-based ILE, olive oil-based ILE was less toxic to lymphocytes, increased ROS production by neutrophils, and did not affect neutrophil viability compared with soybean oil-based ILE [22,47]. Similar findings were reported in another study of isolated human immune cells, where olive oil-based ILE had no effect on lymphocyte proliferation, while soybean oil-based ILE inhibited lymphocyte proliferation [23]. With regard to immune cell function, one study reported that olive oil-based ILE did not significantly affect neutrophil function, including intracellular calcium or elastase release, oxidative burst, chemotaxis, adhesion molecule or leukotriene generation, and phagocytic activity [7]. Another study reported that both olive oil-based and soybean oil-based ILEs induced a significant increase in hydrogen peroxide production (respiratory burst) by neutrophils compared with incubation with no ILE or with MCT/LCT ILE [25]. Respiratory burst is a critical component of the killing function of neutrophils; thus, this study demonstrates that olive oil-based ILE does not appear to diminish neutrophil function. Although it is not clear that olive oil-based ILE improves immune function, the evidence available from in vitro and in vivo studies suggests that olive oil-based ILE preserves existing immune function.

In rats, ILEs have been shown to disrupt bacterial clearing by mononuclear phagocytes [28]. Compared with soybean oil-based ILE and MCT/LCT-based ILE, olive oil-based ILE resulted in less disruption of bacterial clearing despite there being no difference between groups in prostaglandin E_2_ plasma levels [28]. Compared with fish oil-based ILE, structured lipids, MCT/LCT, and soybean oil-based ILE, olive oil-based ILE was the least likely to impair the pneumococcal elimination capacity of isolated human neutrophils [28]. In mouse models of inflammation (trauma-induced cremaster muscle inflammation and lipopolysaccharide-induced inflammation) and lethal endotoxemia, olive oil-based ILE blocked leukocyte recruitment (most likely through modulation of adhesion molecules) and increased survival compared with soybean oil-based ILE and fish oil-based ILE formulations [27]. 

In a study comparing the effects of olive oil-based ILE to soybean oil-based ILE on leukocyte counts in critically ill adults, olive oil-based ILE was associated with an increase from baseline in leukocyte count, whereas soybean oil-based ILE was associated with a decrease from baseline in leukocyte count [26]. The exact mechanism for this effect was not clear; however, it may be possible that the soybean oil-based ILE suppressed the inflammatory response. Not all studies have reported a difference between olive oil-based ILE and soybean oil-based ILE. Two studies, one in healthy adults and the other in surgical intensive care unit (ICU) patients, reported no significant differences in granulocyte phagocytosis, monocyte phagocytosis, granulocyte ROS generation, and monocyte ROS between the olive oil-based ILE and soybean oil-based ILE groups [24,40]. Similar findings were reported in adult patients in the surgical ICU [40]. Importantly, it cannot be ruled out that the differences between these studies may reflect differences between the immune systems of the subjects (i.e., critically ill patients vs. healthy adults).

### 3.2. Inflammation

Compared with other ILEs, there is limited and conflicting evidence to support the benefits of olive oil-based ILE on inflammatory marker profiles. In vitro studies using isolated human peripheral blood mononuclear cells (PBMCs) or polymorphonuclear cells (PMNs) from healthy volunteers revealed that olive oil-based ILE appeared to be more neutral in its effects on inflammatory eicosanoid or cytokine production compared with soybean oil-based, MCT/LCT, or fish oil-based ILEs [7,31,32]. Olive oil-based ILE was not associated with increases in leukotriene B_4_ or prostaglandin-E_2_ production [7,31], or with suppression of tumor necrosis factor α (TNFα), interleukin (IL)-1β, or IL-8 [7,32] levels. These properties of olive oil-based ILE may be advantageous in patients where immune suppression may be detrimental (e.g., ICU patients) [39].

#### 3.2.1. Inflammation Marker Profiles in Adult Clinical Studies

Most adult clinical studies were small and have reported no significant differences between the effects of olive oil and other ILEs on inflammatory marker profiles. In healthy adults, no differences between OO, SO, lipid-free PN, and saline were noted for TNFα, IL-6, or C-reactive protein (CRP).

**Figure 3:**
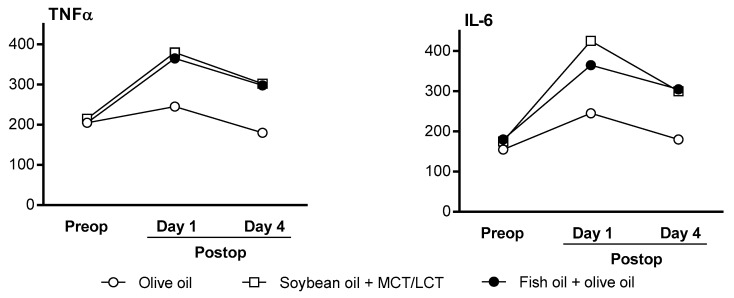
TNFα and IL-6 levels in adult patients undergoing major abdominal surgery (*N* = 52). Reprinted from Demirer, S.; et al. Effects of postoperative parenteral nutrition with different lipid emulsions in patients undergoing major abdominal surgery. *Annals Surg Treat Res* **2016**, *91*, 309–315. CC BY 4.0 [34]. IL-6—interleukin 6; MCT/LCT—medium-chain triglycerides/long-chain triglycerides; postop—postoperative; preop—pre-operative; TNFα—tumor necrosis factor α.

In surgical patients, most studies have not reported differences in inflammatory marker profiles between ILEs. In a recent study comparing soybean oil-based + MCT/LCT (75% soybean oil-based + 25% MCT/LCT) ILE, olive oil-based ILE, and olive oil + fish oil (85% olive oil-based ILE + 15% fish oil) ILE in patients with cancer who had undergone abdominal surgery, TNFα and IL-6 levels were similar between groups at baseline; however, the postoperative increases in TNFα and IL-6 were lower in the olive oil-based ILE group compared with the other groups (Figure 3) [34]. Another study in patients undergoing abdominal surgery reported no differences between groups in CRP [33]. In a study of surgical patients, there was no difference in plasma TNFα, CRP, or IL-6 levels between the olive oil-based ILE and soybean oil-based ILE groups [40]. Similar findings for CRP were reported in another study [20]. In a large randomized controlled trial of surgical patients (*N* = 458), olive oil was associated with a significant decrease in IL-6 levels compared with soybean oil [20]. Thus, the lack of differences noted in earlier studies may reflect a lack of statistical power.

In adults with severe burns receiving an olive oil-based ILE versus a MCT/LCT-based ILE, TNFα decreased significantly from baseline in the olive oil group but not in the MCT/LCT group. IL-6 and IL-10 also decreased from baseline in the olive oil group; however, the decrease was not significant [35]. No between-group differences were noted.

In a study of patients receiving long-term olive oil-based ILE, no differences in TNFα or IL-6 levels between baseline and 3 months were observed [39]. Another study of patients receiving long-term olive oil-based ILE reported that TNFα was 3.6-fold higher in patients compared with healthy controls (likely reflective of the disease), but no differences in IL-10 between groups were noted [37]. The authors also reported that no differences in leukocyte activation, adhesion molecule expression, degranulation markers, or ROS production were noted between the patient and control groups [37]. Further, another study of patients receiving long-term PN with olive oil-based ILE reported that there were no differences in CRP levels between healthy controls and those receiving PN [36].

#### 3.2.2. Inflammation Marker Profiles in Pediatric Clinical Studies

Only one study has examined the effects of ILE on inflammatory marker profiles in pediatric patients [42]. Premature neonates (<32 weeks gestational age and <1500 g) were randomized to either an olive oil-based ILE or a soybean oil-based ILE within the first 48 h of life. Blood samples were collected at baseline and at 14 days, and the PBMCs were isolated and cultured for 48 h in medium only or in the presence of anti-CD3 antibodies. Anti-CD3-stimulated IL-6 increased significantly in the soybean oil group compared with the olive oil group [42]. TNFα and IL-10 were not different between groups.

### 3.3. Infections

Infections in patients receiving PN remain a significant concern. Duration of total PN has been identified as one of the strongest predictors of nosocomial infections in adult [48] and pediatric patients [49] receiving PN. Currently it remains unclear as to whether or not olive oil-based ILE is associated with lower infection rates, with small studies showing no difference between groups. However, in a large randomized controlled trial, olive oil-based ILE was clearly associated with fewer infections compared with a soybean oil-based ILE [20].

#### 3.3.1. Infection Rates in Adult Clinical Studies

**Figure 4:**
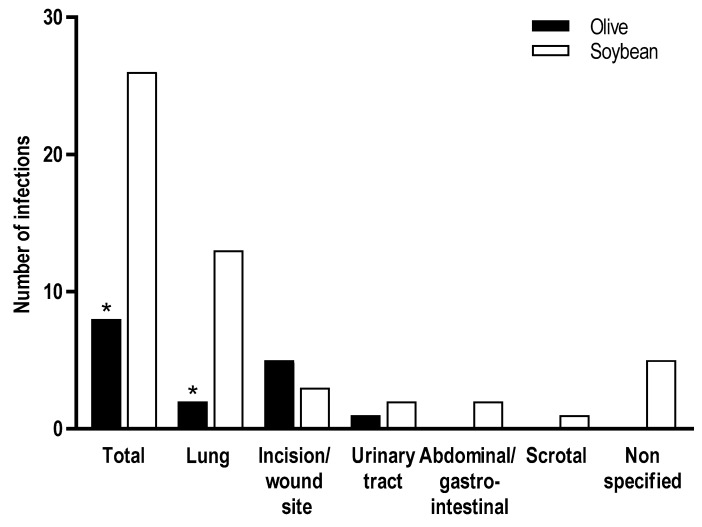
Infection rates in adult surgical patients (*N* = 458) [20]. * *p* < 0.05.

Only one study has reported the effects of ILE on sepsis rates [33]. No significant differences in sepsis rates between olive oil and olive + fish oil groups were noted; however, a significantly lower rate of infections was noted for the olive + fish oil group [33]. In the olive oil group, infection types included respiratory (*n* = 3), abdominal (*n* = 3), urinary tract (*n* = 4), and surgical incision (*n* = 1), while in the olive + fish oil group, infections included abdominal (*n* = 1), surgical wound (*n* = 1), and blood stream infection (*n* = 1) [33]. In several studies, no significant difference between olive oil and soybean oil groups in infection rates was noted; however, these studies were likely underpowered to detect significant differences [26,38,39,40]. In the largest study to date, Jia and colleagues reported that olive oil-based ILE was associated with a significantly lower infection rate compared with soybean oil-based ILE (Figure 4) [20].

#### 3.3.2. Infection Rates in Pediatric Clinical Studies

Several studies, predominantly in preterm neonates, have reported no significant differences in sepsis rates between olive oil-based and soybean oil-based ILEs [41,43,44,45,46].

## 4. Lipid Peroxidation

Oxidative stress is an important mechanism that may contribute to the pathogenesis of inflammation [6]. The fatty acid composition of ILEs (saturated, monounsaturated, and polyunsaturated) results in differential lipid peroxidation; therefore, it has been proposed that ILEs that are high in saturated fatty acids or MUFAs (like olive oil) may be at lower risk of lipid peroxidation [6]. Increased numbers of double bonds, as found in PUFAs, may increase the risk of lipid peroxidation [50]. Additionally, ILEs with higher levels of α-tocopherol may also be more resistant to lipid peroxidation [51,52]. However, paradoxically, excessive/high levels of α-tocopherol may be pro-oxidant [53], thus it is important to ensure that the appropriate amount of α-tocopherol is present in the ILE. Notably, Xu and colleagues found no correlation between the amount of vitamin E present in lipid emulsions and lipid peroxidation [54]; however, the production of hydroperoxides was lowest with olive oil-based ILE and highest with 100% fish oil-based ILE [54].

Studies examining the effects of different ILEs or their main fatty acid constituents suggest that olive oil and its primary constituent, oleic acid, is associated with less lipid peroxidation compared with other ILEs (Table 3) [31,50,55,56,57]. In an in vitro study comparing the effects of fatty acids on ROS production in cultured human colonic cells, docosahexaenoic acid, an important constituent of fish oil, induced a 429% increase in ROS production compared with 6% induced by oleic acid [55]. In mice fed fatty acids (docosahexaenoic acid, oleic acid, or linoleic acid) or oils (fish, olive, or soy), fish oil and docosahexaenoic acid induced ~3-fold increases in postprandial thiobarbituric acid reactive substances (TBARS), a marker of lipid peroxidation [56]. In contrast, olive oil and oleic acid induced modest increases (~0.5-fold) in postprandial TBARS levels [56]. Furthermore, linoleic acid, the primary constituent in soybean oil, resulted in a ~2-fold increase in TBARS levels, while soybean oil induced a ~0.5-fold increase [58]. Lastly, an in vitro study using PBMCs harvested from healthy, fasted volunteers revealed that incubation with olive oil-based or soybean oil-based ILE did not significantly affect lipid peroxide production in mononuclear cells or neutrophils compared with no ILE (control) [31]. However, both Smoflipid and Omegaven induced significant increases in lipid peroxide production in mononuclear cells (2.5-fold and >5-fold, respectively) and neutrophils (2.5-fold and >5-fold, respectively) compared with control [31].

In contrast, numerous clinical studies have investigated markers of oxidative stress including total antioxidant status (TAS), total antioxidant capacity, TBARS, F2-isoprostane, vitamin Eα-tocopherol levels, pentane, or malondialdehyde (MDA). Most studies have reported no differences in oxidative stress markers between olive oil-based and soybean oil-based [20,38,40,43,59,60,61], MCT/LCT [60,62,63], or fish oil-based [64,65] ILE (Table 3). Furthermore, a systematic review of studies in hospitalized pediatric patients suggested that available studies do not support one ILE over another with regard to benefits on oxidative stress [66].

In adult patients after abdominal surgery, TBARS were significantly lower in the olive oil group compared with MCT/LCT + LCT and olive oil + fish oil groups, an effect that was maintained after adjusting for multiplicity [34]. In adults receiving long-term PN with an olive oil-based ILE, malondialdehyde levels did not increase from baseline to 3 months, suggesting that long-term exposure to olive oil-based ILE does not increase oxidative stress [39]. Similarly, another study reported that although adult patients receiving long-term PN with an olive oil-based ILE had increases in oxidized glutathione compared with healthy control subjects, no differences between groups were noted for lipid peroxidation markers and protein carbonyls [36]. Similarly, in children requiring long-term PN, olive oil-based ILE was associated with significantly lower peroxidation products compared with soybean oil-based ILE [51].

## 5. Metabolic Effects

### 5.1. Lipid Metabolism

Administration of PN is associated with increases in plasma cholesterol and triglyceride levels [67]. In the short-term, this transient increase in serum lipid parameters is of less concern than for patients receiving long-term PN. There is evidence to suggest that olive oil-based ILE may have beneficial effects on cholesterol levels, whereas the relationship between olive oil-based ILE and serum triglyceride levels is less clear (Table 4). In guinea pigs, chronic administration of ILEs (10 days) resulted in increased triglyceride levels; however, the levels were significantly lower in soybean oil-based, olive oil-based, and 100% fish oil ILE groups compared with Smoflipid or control (diet) groups [5]. Furthermore, small clinical studies in pediatric and adult populations have shown that olive oil-based ILEs are safe and have limited effects on lipid profiles when used for long-term PN [39,51].

Notably, most studies failed to report the normal reference ranges for lipid markers; however, using the National Cholesterol Education Program Adult Treatment Panel (NCEP-ATP) as a general guide (fasted state), most lipid profiles remained within normal ranges [68]. However, it should be noted that lipid levels measured in patients receiving PN represent the “fed” and not “fasting” state. Therefore, increases in lipid levels above the NCEP-ATP ranges may not represent a true elevation in lipid levels.

#### 5.1.1. Plasma Lipid Levels in Adult Clinical Studies

In healthy adults, plasma triglyceride levels increased significantly in both the olive oil-based and soybean oil-based groups compared with the lipid-free PN and saline treatment groups [24]. In contrast, no differences between treatment groups were noted for total cholesterol (TC), high-density lipoprotein (HDL), or low-density lipoprotein (LDL) between the groups [24]. Despite these significant differences, all plasma lipid values remained largely within normal ranges (fasted state). In this particular study, it should also be noted that the no lipid and saline groups would represent a fasted state; and therefore, comparisons between the ILE groups (fed state) and the no lipid and saline groups (fasted state) should take this into consideration.

In adult patients with severe burns, TC levels increased significantly in response to olive oil-based ILE but remained within the normal range [35]. Triglyceride levels also increased significantly in response to both olive oil-based and soybean oil-based ILEs, exceeding the normal range, with no between-group differences noted.

In adult patients with sepsis or septic shock, both TC and triglyceride levels remained within the normal range after administration of olive oil-based ILE [69]. Further, in adult trauma patients, triglyceride levels were not different between patients receiving olive oil-based ILE and patients receiving no lipid or soybean-based ILE [70].

In adult patients after abdominal surgery, one study reported that in the olive oil group, TC, HDL, LDL, and very low-density lipoprotein (VLDL) levels decreased from baseline, while in the soybean oil group these same parameters increased, with no significant differences between olive oil-based and soybean oil-based ILEs [38]. Another study reported no change from baseline in TC, HDL, LDL, or triglyceride levels after administration of olive oil-based ILE [71]. All lipid levels remained within the normal range [71]. In contrast, another study reported that triglyceride levels increased significantly in both the olive oil-based and fish oil-based ILE groups [72]. Triglyceride levels were higher in the olive oil group and exceeded the normal ranges by Day 2, and the difference between groups was significant [72].

Lastly, in studies of malnourished adult patients, fewer patients receiving olive oil-based ILE experienced deterioration of triglyceride levels compared with patients receiving soybean oil-based ILE [73]. Deterioration was defined as a patient who moved to a more abnormal category after starting PN (categories: within normal limits, elevation up to 2 × upper limit of normal [ULN], and elevation > 2 × ULN), while in adult patients receiving long-term PN, no changes from baseline in TC, HDL, LDL, or triglyceride levels after administration of olive oil-based ILE were noted [36,39].

#### 5.1.2. Plasma Lipid Levels in Pediatric Studies

In children receiving long-term (mean 34 months) PN, olive oil-based ILE was associated with reductions in TC, HDL, LDL, and triglycerides compared with soybean oil, which increased these lipid levels [51]. The differences between groups were significant for TC and LDL. In children with intestinal failure, TC remained within normal limits after administration of olive oil ILE [75].

In preterm neonates, one study reported that olive oil and MCT/LCT were associated with significant increases in triglyceride levels [63]; however, the levels remained within the normal range. Another study reported that olive oil-based ILE maintained HDL levels compared with MCT/LCT; nevertheless, LDL levels were significantly higher for olive oil-based than for soybean oil-based or MCT/LCT ILEs [46]. A third study reported no significant differences in triglycerides, TC, HDL, or LDL between olive oil-based and soybean oil-based ILE; however, VLDL levels were significantly lower in the olive oil-based ILE group [41]. All levels were within normal ranges. Lastly, a fourth study reported no significant differences between olive oil-based ILE and soybean oil-based ILE for TC and triglycerides, and all levels were within normal ranges [43]. Investigation of the metabolism of fatty acids to acylcarnitines indicated that free carnitine, hexanoyl carnitine, and medium-chain fatty acid carnitine levels were significantly lower in the olive oil-based ILE group compared with the soybean oil-based ILE group [41]. These findings suggest that soybean oil-based ILE may impair intramitochondrial metabolism of fatty acids.

### 5.2. Glucose Metabolism

Disturbances of glucose metabolism are common in critically ill patients receiving PN, and this reflects both the dextrose load and possibly the fatty acid composition of the ILE. However, few studies have investigated the direct effects of ILE on glucose metabolism. In normal healthy adults, significant increases in plasma glucose, insulin, and C-peptide were observed after infusion of lipid-free PN, olive oil-based ILE, and soybean oil-based ILE compared with infusion of saline [24]. No significant differences between the lipid-free, olive oil-based ILE, and soybean oil-based ILE groups were noted. These results would suggest that the changes in glucose metabolism likely reflect the dextrose load rather than specific effects of the ILE. In contrast, in preterm infants, soybean oil-based ILE significantly increased gluconeogenesis and glucose production and significantly decreased glycogenolysis compared with glucose, glycerol, and olive oil-based ILE [76]. Olive oil-based ILE did not significantly affect any of these measures. Furthermore, the lack of effect of glycerol on these measures suggests that the fatty acids contained in soybean oil-based ILE may play a role in regulating these processes. The authors suggested that the differences between the ILEs may be useful clinically in that soybean oil-based ILE may be valuable in treating/preventing the hypoglycemia observed during the first few days after birth, while olive oil-based ILE may be beneficial in preventing/treating hyperglycemia [76]. 

### 5.3. Emerging Issues Associated with the Fatty Acid Composition of ILEs

The unique fatty acid compositions of different ILEs impact fatty acid metabolism and as a result may have unintended consequences. As shown in Figure 2, metabolism of fatty acids relies on a few key enzymes, and therefore competition between the fatty acids for these enzymes can impact the fatty acids available for key physiological processes. In preterm neonates, who have no or limited stores of fatty acids, this may be a significant issue as docosahexaenoic acid and arachidonic acid are important for normal brain development. Supplementation with docosahexaenoic acid downregulates the production of both arachidonic acid and docosahexaenoic acid [77,78], and there is evidence to suggest that the ratio of docosahexaenoic acid to arachidonic acid is a key determinant of ensuring adequate supply of both fatty acids [78,79]. Similar to the ratio in breast milk, the appropriate ratio in enteral formulations is approximately 1:2 (docosahexaenoic:arachidonic acid) [78,79]. More recently it has been shown that fish oil-based ILEs (docosahexaenoic:arachidonic acid ratio 1:1) may cause increased rates of retinopathy of prematurity (ROP) compared with olive oil-based ILE (docosahexaenoic:arachidonic acid ratio 1:1.7) [80]. Further studies are needed to elucidate the optimal ratio and concentration of fatty acids in ILE utilized in the preterm neonate population.

In the past 40 years, PN has been infrequently linked to the development of essential fatty acid deficiency (EFAD) and, in the setting of malnutrition, EFAD can occur quickly owing to the lack of essential fatty acid stores. Essential fatty acids are found in high levels in soybean oil, and thus the use of soybean oil-based ILE has been effective in preventing EFAD. With the advent of newer ILEs, such as olive oil-based and fish oil-based ILEs, the potential for EFAD has increased. 

Olthof and colleagues recently published their findings regarding the use of an olive oil-based ILE in patients requiring long-term PN [37]. The study reported that there was no clinical or biochemical evidence of EFAD in patients who had received olive oil-based ILE at least 5 times per week for a period of at least 3 months [37]. The results of this study confirm that, if used daily at the recommended lipid dose, olive oil-based ILE provides sufficient essential fatty acids to prevent EFAD; however, in patients who are receiving lower than recommended doses, e.g., twice-weekly infusions instead of daily, patients may be at risk of EFAD.

## 6. Liver Function

Liver function in patients receiving PN remains a clinical concern for physicians [81,82,83]. Some patients receiving PN may develop liver dysfunction characterized by steatosis and cholestasis. This liver disease is referred to as PN-associated liver disease (PNALD) or intestinal failure-associated liver disease (IFALD) and may progress to steatohepatitis (nonalcoholic-associated steatohepatitis [NASH]), cirrhosis, and liver failure. The etiology of liver dysfunction remains elusive; however, several mechanisms have been proposed [5,44,84,85]:

- Impaired hepatic secretion of fatty acids and triglycerides as VLDL
- Increased synthesis of hepatic triglycerides due to increased intake of n-6 PUFAs and low intake of n-3 PUFAs
- Impaired hepatobiliary secretion leading to cholestasis, possibly resulting from phytosterols present in lipid emulsions and competition of transport owing to differences in phytosterol content
- Impaired hepatobiliary function due to endotoxin entry into the portal circulation or sepsis
- Modulation of oxidative stress and inflammation by peroxidation of PUFAs, increased intake of n-6 PUFAs, and differences in α-tocopherol content
- Lack of enteral nutrition and enteral-stimulated gut growth factors, which may in turn lead to alterations in the gut microbiome.

Although liver disease can occur in adults, children and especially infants are most at risk of developing cholestasis and overt liver disease [82]. The increased risk of IFALD in infants may result from the immature liver development/function along with the use of PN incorporating high lipid doses. In infants, risk factors for IFALD relate to both patient characteristics and management of the intestinal failure. Patient-dependent risk factors include age, degree of liver maturation (prematurity), cause of intestinal failure, site and frequency of infection (gastrointestinal tract, central venous catheter), small-bowel bacterial overgrowth, and enteral feed tolerance [86]. Treatment-related risk factors include the composition of PN [86], its mode of administration (continuous/cyclical) [86], the duration of PN dependency [57,86], the surgical interventions and their anatomical consequences (intestinal obstruction, disruption of the enterohepatic circulation, resection of the terminal ileum or the ileocecal valve) [86], and the use of antibiotics (liver/renal toxicity) [86]. Imbalance (deficiency/excess) of parenteral nutrients has also been implicated in IFALD, and almost all of the components may be possible causative or aggravating agents [86].

We identified 24 studies [20,33,35,38,39,41,43,44,45,46,51,61,64,71,72,73,75,85,87,88,89,90,91,92,93] and three meta-analyses [66,81,83] that investigated the effects of olive oil-based ILE on liver function (Table 5). Findings have varied across studies, and no clear pattern of effect of olive oil-based ILE on markers of liver function (sometimes called liver function tests) such as alanine aminotransferase (ALT), aspartate aminotransferase (AST), and bilirubin (total or conjugated) or on biliary tract function markers such as alkaline phosphatase (ALP) and gamma-glutamyl transpeptidase (GGT) have been discerned. Importantly, while statistically significant differences between olive oil-based ILE and other ILEs were noted in many studies (Table 5), the majority of studies reported hepatobiliary functional marker levels that were within the normal ranges or within 1.5 × ULN [94]. Slight elevations (up to 1.5 × ULN) do not necessarily indicate the presence of liver disease [94]. Thus, these statistically significant differences between ILEs need to be interpreted with caution as they may not be clinically important. Most studies in adults, preterm neonates, and children suggest that olive oil-based ILE is safe and not associated with adverse effects on hepatobiliary function.

### 6.1. Hepatobiliary Function in Adult Studies

Most studies of adult patients have demonstrated that, in general, olive oil-based ILE is not associated with adverse effects on the hepatobiliary system. In adults post-abdominal surgery, four small studies reported that ALP, AST, and ALT levels remained or decreased to normal ranges (or less than 1.5 × ULN), suggesting that olive oil-based ILE preserved liver function in these patients [33,38,71,72]. Two of three studies reported that GGT was within the normal range (or less than 1.5 × ULN) [33,38], while the third study reported that although GGT levels were higher than the normal range, they did not change from baseline [71], i.e., olive oil-based ILE did not worsen pre-existing biliary dysfunction. Of two studies reporting on total bilirubin, one reported that olive oil-based ILE significantly decreased total bilirubin levels from baseline, whereas the other reported no change; however, the levels were within normal limits [38,71]. Similar findings for liver function tests have been reported for severely malnourished adult patients [73], adult patients receiving long-term PN [39,90], adult patients with esophageal cancer [92], and adult patients with severe burns [35].

In another study of adult patients receiving PN for longer than 6 months, transitioning patients from soybean oil-based ILE to olive oil-based ILE did not result in changes to biliary outflow efficiency, and imaging revealed no abnormalities at baseline or at the end of the study [90].

**Figure 5:**
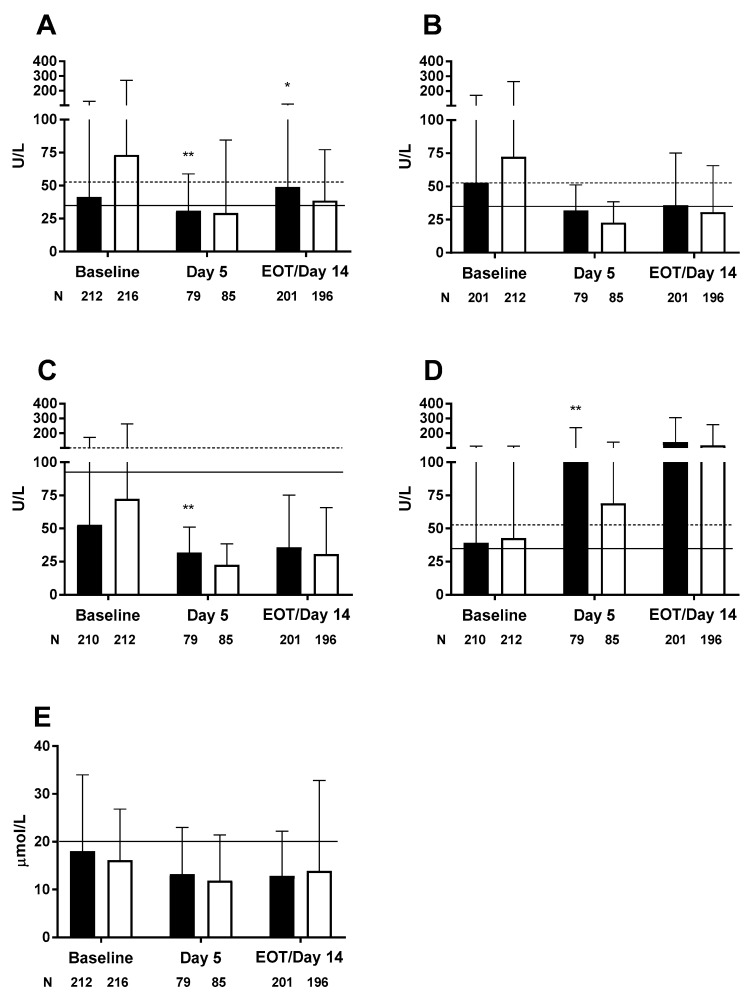
Liver enzymes in adult surgical patients; (**A**) alanine aminotransferase, (**B**) aspartate aminotransferase, (**C**) alkaline phosphatase, (**D**) gamma-glutamyl transpeptidase, and (**E**) total bilirubin. Black bars denote olive oil-based ILE, white bars denote soybean oil-based ILE, solid horizontal line denotes upper limit of normal range, dotted horizontal line denotes 1.5 × ULN. Values up to 1.5 × ULN are not considered clinically meaningful [20]. * *p* < 0.05, ** *p* < 0.005. EOT—end of therapy; ILE—intravenous lipid emulsion; ULN—upper limit of normal range.

In the largest study to date (*n* = 458), adult surgical patients were randomized to either olive oil-based ILE (*n* = 226) or soybean oil-based ILE (*n* = 232) for a minimum of 5 and a maximum of 14 days [20]. In this large randomized controlled trial, liver enzymes were generally within normal limits (Figure 5). No incidences of clinically relevant liver disease were observed and no lipid dose reductions were required [20]. 

Most recently, a study of adult patients receiving PN for >12 months due to intestinal failure reported that olive oil-based ILE significantly reduced bilirubin levels compared with soybean oil-based, MCT/LCT, and fish oil-based ILE [93]. No differences between the soybean oil-based, MCT/LCT, or fish oil-based ILE were observed for any marker of hepatobiliary function [93].

Few studies have directly compared the effects of olive oil-based ILE with fish oil-based ILE; of those that have, most reported no significant differences between groups in most LFT results [33,44,64]. Only one study reported significant differences in LFTs between olive oil-based and fish oil-based ILE; however, levels in both groups were within the normal range [74]. Additionally, long-term PN (1 year) with olive oil-based ILE significantly reduced bilirubin and GGT levels [93]. In contrast, fish oil-based ILE had no effect on bilirubin or any other LFTs [93].

### 6.2. Hepatobiliary Function in Pediatric Studies

In preterm neonates, most studies have reported no adverse effects of olive oil-based ILE on liver function. Most studies reported that conjugated bilirubin, currently the best marker of cholestasis, was within the normal range [41,43,44,45,46,64,87].

One study reported that conjugated bilirubin levels were substantially above the normal range in preterm neonates (<30 weeks gestational age) [64]. Notably, no differences between groups were observed at Day 8 (olive: 6.85 ± 5.12 µmol/L vs. fish: 5.53 ± 3.04 µmol/L) [64]. ALT and AST were within the normal range in all studies that assessed these liver enzymes [43,44,45,46,61,64,87]. In contrast, ALP and GGT were well above the normal range after PN administration in all but one study [44] that assessed these biliary tract function markers [43,45,46,64,87].

In children aged greater than 1 year, olive oil-based ILE has not been associated with adverse effects on liver function. In children undergoing bone marrow transplantation, small elevations in ALT, AST, ALP, and GGT were reported; however, none of the changes were statistically significant and values were within normal ranges [62]. Total bilirubin decreased slightly from baseline; however, this effect was not statistically significant. In children with intestinal failure who were stabilized on long-term PN, liver function tests remained within normal ranges (or within 1.5 × ULN); however, liver biopsies revealed some degree of fibrosis in five of eight patients [75].

Another study of children receiving long-term PN (with olive oil-based ILE or soybean oil-based ILE) reported that liver enzymes increased from baseline to Day 60; ALP and total bilirubin levels were substantially greater than normal, while ALT, AST, and GGT were within normal or 1.5 × ULN [51].

Lastly, meta-analyses and systematic literature reviews have reported that there is no evidence to suggest that there are benefits of one lipid emulsion over another in terms of effects on liver enzymes or total bilirubin [66,81,83].

## 7. Endothelial Function

Endothelial cells play a critical and multifunctional role in the maintenance of vascular function, including necessary actions such as preserving vascular tone, blood fluidity, and vascular permeability [95,96]. Endothelial cells also regulate inflammatory responses as well as modulating hemostasis/thrombosis, fibrinolysis, and angiogenesis [97]. Endothelial cells are critical to an effective immune response as they regulate the migration of leukocytes and their transition from the blood to the site(s) of infection [98]. In the setting of cardiovascular disease as well as metabolic disorders, significant research has demonstrated that free fatty acids may directly contribute to endothelial dysfunction [97]. In the setting of PN, this may have important implications for patients who are already hyperinflamed or immunocompromised.

There is limited evidence to suggest that ILEs may exert direct effects on endothelial function [52,99,100,101]. In porcine coronary artery rings, soybean oil-based and olive oil-based ILEs did not reduce bradykinin-induced relaxation of endothelial cells, while MCT/LCT and Smoflipid did [101]. In normotensive, healthy adults, soybean oil-based ILE induced a rapid and sustained increase in blood pressure and decreased endothelial function compared with olive oil-based ILE and lipid-free ILE [24]. Infusion of olive oil-based ILE did not alter flow-mediated dilatation, while infusion of soybean oil-based ILE significantly decreased flow-mediated dilatation from baseline to 4 h and 24 h [24].

In cultured human aortic endothelial cells, the effects of different ILEs on fatty acid uptake and incorporation, integrity, and inflammatory activation varied depending on the ILE used [102]. Fatty acid uptake by endothelial cells was shown to be dose dependent and was lowest in the soybean oil-based ILE group and highest in the olive oil-based ILE group. Regarding endothelial cell apoptosis/necrosis, olive oil-based ILE increased endothelial cell viability, fish oil-based ILE reduced cell viability, and soybean oil-based ILE had no effect. The effect of ILEs on the proinflammatory response in endothelial cells was assessed by their effect on lipopolysaccharide-induced surface expression of intracellular adhesion molecule-1. Although all three ILEs suppressed the endothelial cell inflammatory response, the fish oil-based ILE may be the most potent, and its inhibitory effects were consistent with other studies using fish oil-based ILEs and omega-3 fatty acids [102].

**Figure 6:**
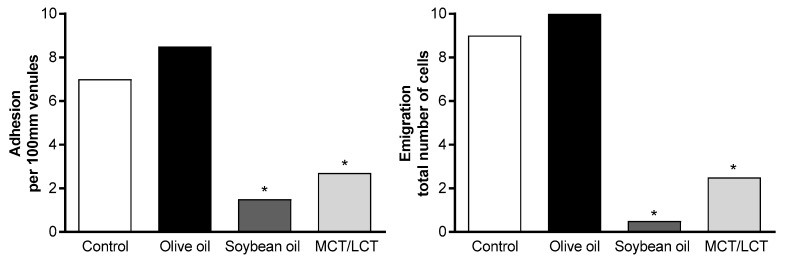
Adhesion and emigration of LPS-induced leukocytes in a rat model. Reprinted from Demirer, S.; et al. Effects of postoperative parenteral nutrition with different lipid emulsions in patients undergoing major abdominal surgery. *Annals Surg Treat Res* **2016**, *91*, 309–315. CC BY 4.0. * *p* < 0.05. LPS—lipopolysaccharide; MCT/LCT—medium-chain triglycerides/long-chain triglycerides.

Transmigration of leukocytes from the blood to infected/inflamed tissues is regulated by the endothelial cells through the expression of cytokines and adhesion molecules [103]. An in vitro/in vivo study investigating the effects of olive oil-based, soybean oil-based, and MCT/LCT ILEs revealed that only the olive oil-based ILE preserved adhesion and emigration of leukocytes, thereby maintaining transmigration, compared with soybean oil-based and MCT/LCT ILEs (Figure 6) [7].

## 8. Clinical Outcomes

The goal of PN is to provide nutrition to patients who would otherwise not receive their daily calories and nutrients. In addition to their effects on cellular, metabolic, and liver function markers, some studies have evaluated the effects of ILEs on morbidity and mortality. Currently there is limited evidence that olive oil-based ILE offers any significant benefit over other ILEs on morbidity or mortality outcomes. However, it should also be noted that other, newer ILEs such as those containing fish oil also have not been shown to consistently confer benefits on these important outcomes [104,105].

In most studies of adult patients, no differences between ILEs were reported for mortality, length of hospital stay, length of ICU stay, termination of mechanical ventilation, or duration of mechanical ventilation [33,40,69,106]. However, one study did report a significant benefit of olive oil-based ILE on length of ICU stay and duration of mechanical ventilation compared with patients receiving no ILE [70]. A meta-analysis reported that olive oil-based ILE was not associated with significant reductions in mortality or length of ICU stay compared with soybean oil-based or MCT/LCT ILEs [107]. In contrast, olive oil-based ILE was found to significantly reduce the duration of mechanical ventilation (risk ratio −6.47; 95% confidence interval −11.41, −1.53; *p* = 0.01) compared with soybean oil-based or MCT/LCT ILEs [107]. The findings from this meta-analysis should be interpreted with caution as they are based on the results of only two studies.

In pediatric patients, the evidence is less clear, with conflicting results across studies. One study of preterm, very low birth weight neonates (<1500 g) reported no differences between olive oil-based and soybean oil-based ILEs in duration of mechanical ventilation, bronchopulmonary dysplasia, necrotizing enterocolitis, or retinopathy of prematurity [42]. Another study of preterm neonates (≤34 weeks) reported a significant benefit of olive oil-based ILE compared with soybean oil-based ILE on bronchopulmonary dysplasia and duration of mechanical ventilation [43]. In a study of preterm very low birth weight (<1250 g) neonates, the combination of olive oil-based ILE plus fish oil (50% + 50%) was associated with a significant reduction in the incidence of ROP that required laser therapy compared with olive oil-based ILE alone; however, there was no difference between treatment groups in ROP grades 1–3, bronchopulmonary dysplasia, necrotizing enterocolitis, sepsis, or length of hospitalization [108]. Lastly, one study of preterm, very low birth weight neonates (500–1249 g) compared five different ILEs [44]. No statistically significant differences between groups (soybean oil-based, olive oil-based, MCT/LCT, MCT/LCT + soybean oil + fish oil, or Smoflipid ILEs) for bronchopulmonary dysplasia, necrotizing enterocolitis, sepsis, or patent ductus arteriosus were observed [44]. 

## 9. Stability

The physicochemical stability of PN solutions is an important consideration as destabilization can lead to catastrophic consequences such as pulmonary emboli. Physical destabilization of a PN solution includes the formation of precipitates as well as an increase in fat globule size to greater than the industry-established 5 micron limit [109]. Physical instability occurs due to the loss of the net negative charge upon the lipid droplets. This primarily results from the introduction of positively charged cations in the form of electrolytes, minerals, or amino acids [110].

The United States Pharmacopeia sets the maximum percentage of fat globules greater than 5 microns (PFAT5) as 0.05% for ILEs, and all commercially available ILEs must meet this standard at the time of manufacture. However, the PFAT5 can increase above the industry standard during the standard hang time of 24 h [111]. Importantly, destabilization of the PN solution cannot be determined in most cases by visual inspection [112].

Several studies have reported that olive oil-based ILE better maintains its physicochemical stability compared with other currently available ILEs [111,113]. Compared with three soybean oil-based ILEs, olive oil-based ILE had the smallest globule size distribution, with the least variation in size between globules [2]. Another study showed that the physicochemical stability deteriorated substantially over time for soybean and soybean/safflower oils compared with MCT/LCT and olive oil-based ILEs [111].

More recently, there has been the development of multi-chamber bag (MCB) formulations of PN that combine dextrose, amino acids, and lipids that allow the mixing of the constituents at the time of use. The benefits of MCBs include the potential for reduced risk of infections occurring during compounding as well as ease of use [20,114]. Furthermore, the use of MCBs eliminates the risk of precipitation or destabilization of the PN solution, which can occur when Y-site administration is used [115]. The stability of the ILE in MCBs was assessed and revealed that no fat particles over 5 microns were observed for either olive oil-based or soybean oil-based ILE [113]. Notably, the tendency for separation of large diameter droplets in the two emulsion systems was different, with the large droplets being located at the top of the bag in the olive oil-based ILE and at the bottom of the bag in the soybean oil-based ILE independent of the calcium content of the PN admixtures. This observation may have important clinical implications, as the large droplets at the top of the bags are less likely to be infused than those at the bottom of the bag. The difference between the two types of PN mixtures could be partly explained by the different glucose content and its effect on the osmolarity, and by the slightly different density of the two emulsions. Another study investigated the stability of a fish oil-containing ILE (Smoflipid) in MCBs and reported that PFAT5 values of all the tested samples were below the USP-specified limits (0.05%) [116]. This was consistent across all study conditions (12 months of storage at 25 or 30 °C, or 6 months of storage at 40 °C) and the maximum value never exceeded 0.016% [116].

## 10. Conclusions

Olive oil-based ILE has been available for clinical use for over 20 years and during that time has proven to be effective at meeting the nutritional needs (including energy and essential fatty acid requirements) of, as well as being well tolerated in, numerous PN-requiring populations. There is evidence to suggest that olive oil-based ILE preserves immune function by supporting the innate immune system, and this is borne out by the lower infection rates seen in a large randomized controlled trial in critically ill patients [20]. Similarly, olive oil-based ILE appears to result in less lipid peroxidation compared with soybean oil-based ILEs, most likely due to its high MUFA and low PUFA content. In most studies, olive oil-based ILE maintained hepatobiliary marker and plasma lipid levels within normal or near normal ranges, and in patients receiving long-term PN, olive oil-based ILE was not associated with increased hepatobiliary or lipid disturbances. No clear differentiation between ILE formulations has been observed for sepsis rates, morbidity, mortality, or for prevention of liver damage. These data would suggest that olive oil-based ILE is a valuable option in various PN-requiring patient populations.

## Figures and Tables

**Table 1:** Fatty acid composition of commercially available parenteral nutrition lipid emulsions.

Constituent	Intralipid	ClinOleic	Lipofundin MCT/LCT	Structolipid	Omegaven	Lipoplus/Lipidem	Smoflipid
Oil Source	100% Soybean	80% Olive 20% Soybean	50% MCT 50% Soybean	36% MCT 64% Soybean	100% Fish	50% MCT 40% Soybean 10% Fish	30% MCT 30% Soybean 25% Olive 15% Fish
**Fatty acid composition, % of total**							
Medium-chain FA							
Caprylic	ND	ND	27.0	14.47	ND	24.18–30.1	16.0–20.5
Capric	ND	ND	17.95	9.34	ND	16.13–19.4	9.85–13.0
Long-chain FA							
Oleic acid	20.92	59.69	11.68	16.55	10.15	7.9–13.44	25.2–30.77
α-linolenic	6.65	1.71	ND	5.72	1.23	2.42–3.41	2.0–2.75
Eicosapentaenoic	ND	ND	ND	NA	19.34	2.75–3.69	2.35–3.03
Docosahexaenoic	0.11	0.06	0.06	0.19	17.67	2.3–2.53	1.73–2.75
Arachidonic	0.18	0.16	0.19	0.24	1.47	0.52–0.66	0.27–0.5
Linoleic	54.68	18.56	28.89	39.18	2.98	20.88–25.72	17.8–21.42
**Phytosterols, mg/L**							
β-sitosterol	302.6	240.6	191.6	240.0	ND	NA	131.6
Campesterol	55.4	13.3	30.9	44.0	1.0	NA	20.5
Stigmasterol	65.1	12.2	46.0	48.8	1.4	NA	18.5
**Tocopherols, µg/mL ± SD**							
α-tocopherol	21.0 ± 0.2	32.0 ± 0.7	132.0 ± 5.6	28.4 ± 1.0	230.0 ± 0.8	177.0 ± 0.7	164.5 ± 2.7
β-tocopherol	3.8 ± 0.7	0.6 ± 0.1	2.1 ± 0.1	1.9 ± 0.0	ND	1.5 ± 0.0	1.5 ± 0.1
γ-tocopherol	108.0 ± 0.9	14.0 ± 0.0	68.0 ± 1.0	68.6 ± 0.7	0.2 ± 0.0	57.0 ± 0.3	29.2 ± 0.6
δ-tocopherol	33.0 ± 0.2	11.0 ± 0.0	21.0 ± 0.2	27.7 ± 0.1	0.0 ± 0.0	69.0 ± 0.3	10.7 ± 0.1

Information taken from [5,11,12,13,14,15,16,17,18,19]. FA—fatty acids; MCT—medium-chain triglycerides; MUFA—monounsaturated FA; NA—not available, ND—not detected; PUFA—polyunsaturated FA; SD—standard deviation; SFA—short-chain FA.

**Table 2:** Effects of olive oil-based intravenous lipid emulsions on inflammation, immune function, and infections.

Study	Population	Intervention and Control (*n*) (Lipid Dose)	Duration	Outcome in Intervention Group
**In vitro and in vivo studies**				
Buenestado et al., 2006 [7]	In vitro: human neutrophils In vivo: rat leukocytes within mesenteric microcirculation	OO SO MCT/LCT In vitro: lipid-free medium In vivo: saline infusion	In vitro: 1 h to 48 h incubation In vivo: 2 h IV infusion	OO had lower impact on neutrophils (in vitro) and leukocytes (in vivo) compared with other ILEs
Buschmann et al., 2015 [27]	In vitro: Murine aortic endothelial cells and bone marrow PMNs In vivo: Mice	OO MCT/LCT SMOF Saline	In vitro: 3 h incubation In vivo: bolus injection (1 to 3 injections)	During systemic inflammation, OO had superior anti-inflammatory properties compared with other ILEs
Cury-Boaventura et al., 2008 [22]	In vivo: human lymphocytes and neutrophils	OO (*n* = 20) Saline (*n* = 3)	6 h IV infusion	Decreased lymphocyte proliferation Promoted lymphocyte necrosis (by lipid accumulation) No effect on the proportion of viable neutrophils
	In vitro: human peripheral white blood cells	OO SO	48 h incubation	No effect on lymphocyte proliferation
Juttner et al., 2008 [25]	In vitro: human neutrophils and monocytes	OO SO MCT/LCT	Incubations up to 1 h	SO and OO (to lesser extent) induced hydrogen peroxide production in neutrophils and monocytes compared with MCT/LCT, which had no effect
Nanhuck et al., 2009 [31]	In vitro: human PMNs and PBMCs	OO SO SMOF FO Saline	18 h incubation	No difference in the production of lipid bodies from stimulated PMNs or PMBCs between the ILE groups Higher production of eicosanoids and lipid peroxides in FO group
Reimund et al., 2004 [32]	In vitro: human PBMCs	OO SO MCT/LCT	24 h incubation	Basal (non-stimulated) PBMC TNFα production decreased significantly for all ILEs in a dose-dependent manner; however, it was most preserved in the OO group compared with SO (*p* = 0.0004) and MCT/LCT (*p* = 0.0483) No effect on IL-6 and IL-8 production was noted for any of the ILEs LPS-stimulated cytokine production was not affected by OO or MCT; however, IL-1 production was significantly inhibited by SO in a dose-dependent manner (*p* = 0.02)
Versleijen et al., 2010 [29]	In vitro: human neutrophils	OO SO MCT/LCT FO SL	1 h incubation	Basal elimination capacity (pneumococcal elimination mean ± SD: 75% ± 3%) decreased significantly for all ILEs; however, it was most preserved in the OO group (70% ± 6%; *p* = 0.045) compared with SO (66% ± 10%; *p* = 0.046), MCT/LCT (47% ± 15%; *p* = 0.028), FO (67% ± 2%; *p* = 0.028), and SL (63% ± 9%; *p* = 0.028)
**Animal studies**				
Garnacho-Montero et al., 2002 [28]	Rats	OO (*n* = 15) SO (*n* = 17) MCT/LCT (*n* = 12) Chow (*n* = 15) Glucose (*n* = 12)	4 day	OO caused less disruption of bacterial clearing
**Adult studies**				
Badía-Tahull et al., 2010 [33]	Gastrointestinal surgery (oncology)	OO (*n* = 14) (0.88 g/kg/day) OO + FO (84.4% + 16.6%) (*n* = 13) (0.88 g/kg/day)	5 day	Significantly fewer infections in the OO + FO group compared with the OO group (3 vs. 11; *p* = 0.007) No differences between groups for C-reactive protein
Demirer et al., 2016 [34]	Abdominal surgery (oncology)	OO {100%} (*n* = 13) (NR) SO + MCT/LCT {75% + 25%} (*n* = 18) (NR) OO + FO {85% +15%} (NR) (*n* = 21)	≥4 day	No significant difference in cytokines (TNFα and IL-6) between groups; however, lower levels were observed for the OO group
García-de-Lorenzo et al, 2005 [35]	Patients with severe burns	OO (*n* = 11) (1.3 g/kg/day) MCT/LCT (*n* = 11) (1.3 g/kg/day)	6 day	Significant reduction in TNFα from baseline for OO Non-significant reduction in other cytokines (IL-6 and IL-10) from baseline No difference compared with MCT/LCT ILE
Jia et al., 2015 [20]	ICU	OO (*n* = 226) (0.8 g/kg/day) SO (*n* = 232) (0.8 g/kg/day)	5–14 day	Fewer infections in OO-based PN group IL-6 decreased in both groups at Day 5 and was undetectable at Day 14/EOT. The difference between groups at Day 5 was significant (*p* = 0.0173) C-reactive protein decreased from baseline in both groups with no differences at any time point
Mateu-de Antonio et al., 2008 [26]	ICU patients	OO (*n* = 23) (0.86 g/kg/day) SO (*n* = 16) (0.91 g/kg/day)	≥5 day	No effect on infection rate, acute-phase proteins, and major health outcomes Higher leukocyte count at end of PN and higher peak leukocyte count in the OO group
Olthof et al., 2013 [36]	Long-term PN	OO (*n* = 20) (NR) Healthy controls (*n* = 21)	≥6 months	No significant difference between groups in C-reactive protein. Values within normal reference range No significant differences between groups in elimination of *Streptococcus pneumoniae* or expression of membrane surface activation markers
Olthof et al., 2016 [37]	Long-term PN	OO (*n* = 30) (0.97 g/kg/day) Healthy controls (*n* = 30)	≥3 months	TNFα production by PBMCs increased 3.6-fold in the OO group compared with controls (*p* < 0.001), while IL-10, C-reactive protein, and membrane activation markers were not different between groups
Onar et al., 2011 [38]	Abdominal surgery (oncology)	OO (*n* = 10) (0.75 g/kg/day) SO (*n* = 10) (0.75 g/kg/day)	7 day	No significant difference in infection rates between OO and SO ILEs
Reimund et al., 2005 [39]	Long-term PN	OO (*n* = 14) (31% of calories)	3 months	No significant modifications in measured inflammatory (e.g., TNFα and IL-6) and immune parameters concentrations
Siqueira et al., 2011 [24]	Healthy subjects	OO (NR) SO (NR) Lipid-free PN Saline	24 h infusion of each intervention (random order) *	No differences in inflammatory markers (TNFα, IL-6, or C-reactive protein) or immune function parameters (granulocyte or monocyte phagocytosis, and granulocyte or monocyte ROS generation) between groups
Umpierrez et al., 2012 [40]	Surgical ICU	OO (*n* = 51) (22 kcal/kg/day) SO (*n* = 49) (22 kcal/kg/day)	Maximum 28 day	No difference in plasma inflammatory markers (C-reactive protein, IL-6, and TNFα), or immune cell function (granulocyte or monocyte phagocytosis, granulocyte or monocyte ROS generation), and similar rates of infections between OO and SO ILEs
**Preterm infant studies**				
Demirel et al., 2012 [41]	≤32 week	OO (*n* = 20) (up to 3 g/kg/day) SO (*n* = 20) (up to 3 g/kg/day)	14 day	No significant differences in sepsis rates between OO and SO ILEs
Gawecka et al., 2008 [42]	<1500 g and <32 week	OO (*n* = 18) (2.7 g/kg/day) SO (*n* = 20) (2.7 g/kg/day)	14 day	Anti-CD3 stimulated IL-6 increased significantly in the SO compared with OO/SO group. No difference in stimulated or unstimulated TNFα and IL-10 between groups
Koksal et al., 2011 [43]	≤34 week	OO (*n* = 32) (up to 3 g/kg/day) SO (*n* = 32) (up to 3 g/kg/day)	7 day	No significant differences in sepsis rates between OO and SO ILEs
Savini et al., 2013 [44]	500–1249 g	OO (*n* = 29) (up to 3 g/kg/day) SO (*n* = 30) (up to 3 g/kg/day) MCT/LCT (*n* = 30) (up to 3 g/kg/day) SO/MCT/FO (*n* = 27) (up to 3 g/kg/day) MCT/SO/FO (*n* = 28) (up to 3 g/kg/day)	21 day	No significant differences in sepsis rates between the 5 tested ILEs
Wang et al., 2016 [45]	<2000 g and <37 week	OO (*n* = 50) (1.45 g/kg/day) SO (*n* = 50) (1.41 g/kg/day)	>14 day	No significant differences in sepsis rates between OO and SO ILEs
Wang et al., 2016 [46]	<2000 g and <37 week	OO (*n* = 50) (1.42 g/kg/day) SO (*n* = 50) (1.39 g/kg/day) MCT/LCT (*n* = 50) (1.30 g/kg/day)	>14 day	No significant differences in sepsis rates between the 5 tested ILEs

* Patients (*n* = 12) received a 24-h infusion of each lipid emulsion (in random order) on 2 consecutive days. CRP—C-reactive protein; FO—fish oil-based ILE; ICU—intensive care unit; IL—interleukin; ILE—intravenous lipid emulsions; LCT—long-chain triglycerides; MCT—medium-chain triglycerides; MCT/LCT—ILEs that combine soybean LCT and MCTs from coconut oil; OO—olive oil-based ILE; PBMCs—peripheral blood mononuclear cells; PMNs—polymorphonuclear cells; PN—parenteral nutrition; ROS—reactive oxygen species; SL—Structolipid; SO—soybean oil-based ILE; SO/MCT/FO—ILE that combines soybean LCT, MCTs, and fish oil; SO/MCT/OO/FO—ILE that combines soybean LCT, MCTs, olive oil, and fish oil; TNFα—tumor necrosis factor alpha.

**Table 3:** Effects of olive oil-based intravenous lipid emulsions on lipid peroxidation.

Study	Population	Intervention and Control (*n*) [Lipid Dose]	Duration	Outcomes
**In vitro studies**				
Watkins et al., 1998 [55]	In vitro: HT-29 human colonic adenocarcinoma cells	Oleic acid Linoleic acid Docosahexaenoic acid Eicosapentaenoic acid Arachidonic acid Control	36 h	ROS production was: oleic acid 6%; linoleic acid 35%, arachidonic acid 94%, eicosapentaenoic acid 40%, and docosahexaenoic acid 429% greater than control
Nanhuck et al., 2009 [31]	In vitro: isolated human PBMCs and PMNs	OO SO FO SMOF All ILEs were delivered as 0.01%, 0.02%, or 0.04%	18 h	In both PMBCs and PMNs, OO and SO consistently showed no effects on LTB_4_, FO dramatically increased LTB_4_ in both LPS-stimulated and unstimulated cells Effects on PGE_2_ were similar, but were not always linear In both PMBCs and PMNs, FO significantly increased lipid peroxide generation, compared with the other ILE and control. SMOF induced a small increase at the highest dose compared with the control, but not the other ILEs
**Animal studies**				
Fuhrman et al., 2006 [56]	BALB/c mice	Oleic acid Linoleic acid Docosahexaenoic acid OO SO FO Saline	2 h	Oxidative stress responses increased after intake of all unsaturated fatty acids and oil supplements. However, FO and docosahexaenoic acid induced the greatest increases compared with saline
Xu et al., 2016 [54]	Guinea pigs	OO SO FO SMOF	10 day	MDA levels were increased in the SO, FO, and SMOF groups, with the highest levels seen in the FO group and the lowest seen in the OO group (OO vs. FO; *p* < 0.05)
**Adult studies**				
Demirer et al., 2016 [34]	Abdominal surgery (oncology)	OO {100%} (*n* = 13) (NR) SO + MCT/LCT {75% + 25%} (*n* = 18) (NR) OO + FO {85% + 15%} (*n* = 21) (NR)	≥4 day	TAS decreased slightly in all groups (*p* = NS) and TBARS increased in all groups, but were lowest in the OO group (*p* ≤ 0.0015) and remained significant after Bonferroni’s was performed
Jia et al., 2015 [20]	ICU	OO (*n* = 226) (0.8 g/kg/day) SO (*n* = 232) (0.8 g/kg/day)	5–14 day	F2-I and MDA were not significantly different from baseline or between groups
Onar et al., 2011 [38]	Abdominal surgery (oncology)	OO (*n* = 10) (0.75 g/kg/day) SO (*n* = 10) (0.75 g/kg/day)	7 day	TBARS increased in both groups, no significant difference between groups
Olthof et al., 2013 [36]	Long-term PN	OO (*n* = 20) (NR) Healthy controls (*n* = 21)	≥6 months	Total glutathione concentration was not different between groups, oxidized glutathione was higher in PN group (*p* < 0.001). Lipid peroxidation products, plasma concentrations of vitamin E, and glutathione were not different between groups. Protein carbonyl levels were below detection limits in both groups
Reimund et al., 2005 [39]	Long-term PN	OO (*n* = 14) (31% of calories)	3 months	Vitamin E and MDA did not change from baseline to 3 months
Umpierrez et al., 2012 [40]	ICU	OO (*n* = 51) (22 kcal/kg/day) SO (*n* = 49) (22 kcal/kg/day)	28 day	Markers of oxidative stress were similar between groups at baseline, Day 3, and Day 7
**Pediatric studies**				
Goulet et al., 1999 [51]	Long-term PN	OO (*n* = 9) (1.92 g/kg/day) SO (*n* = 9) (1.69 g/kg/day)	Mean >30 months	LV-TBARS (*p* = 0.0027), the ratio of LDL-TBARS to LDL (*p* = 0.0262), and the ratio of LV-TBARS to LV (*p* = 0.0146) were significantly increased in the SO group compared with the OO group
Hartman et al., 2009 [62]	Bone marrow transplant	OO (*n* = 15) (1.1 g/kg/day) MCT/LCT (*n* = 13) (1.1 g/kg/day)	14 day	TBARS and vitamin E did not change from baseline and there were no differences between groups
**Preterm neonate studies**				
Deshpande et al., 2014 [64]	<30 week	OO (*n* = 17) (18.45 g/kg/day) SMOF (*n* = 17) (18.25 g/kg/day)	7 day	F2-I did not change from baseline in the OO group and decreased in the FO group. Difference between groups in change from baseline was significant (*p* = 0.0372) Vitamin E increased significantly in both groups (OO *p* = 0.0007, FO *p* = 0.0004), and the change from baseline was significantly higher for FO than for OO (*p* = 0.0091)
Deshpande et al., 2009 [59]	23–28 week	OO (*n* = 24) (1.89 g/kg/day) SO (*n* = 21) (1.89 g/kg/day)	5 day	F2-I decreased significantly in both groups (OO *p* = 0.006, SO *p* = 0.013), but there was no difference between groups in the change from baseline
Koksal et al., 2011 [43]	≤34 week	OO (*n* = 32) (up to 3 g/kg/day) SO (*n* = 32) (up to 3 g/kg/day)	7 day	TAC decreased in both groups from baseline, but there was no difference between groups
Pitkanen et al., 2004 [63]	28–33 week	OO (0.48 g/kg/day) MCT/LCT (0.48 g/kg/day)	3 h *	Pentane levels significantly increased in both groups during PN infusion, difference between groups was not significant
Roggero et al., 2010 [60]	28–33 week	OO (*n* = 12) (up to 3 g/kg/day) SO (*n* = 12) (up to 3 g/kg/day) MCT/LCT (*n* = 12) (up to 3 g/kg/day)	7 day	F2-I and TRAP concentrations were not statistically different within and among the 3 groups at any time of the study. No significant interaction effect between the type of lipid emulsion administered and the repeated values of F2-I and TRAP was found. F2-I values showed a trend to decrease throughout the study in all the 3 groups
Unal et al., 2017 [65]	25–32 week	OO (*n* = 134) (up to 3 g/kg/day) SMOF (*n* = 93) (up to 3 g/kg/day)	Median 7 day	TAC, TOS, and OSI significantly decreased from baseline to Week 3 in both groups (all *p* < 0.001)
Webb et al., 2008 [61]	25 week–7 day	OO (*n* = 39) (23.1 kcal/kg/day) SO (*n* = 40) (24.3 kcal/kg/day)	5 day	F2-I levels were not different between groups at baseline or Day 5

* Patients (*n* = 13) received a 3-h infusion of each lipid emulsion (in random order) on 2 consecutive days. F2-I—F2-isoprostane; LV—low-density lipoprotein + very low-density lipoprotein; MCT/LCT—medium-chain triglycerides/long-chain triglycerides; MDA—malondialdehyde; OO—olive oil; OSI—oxidative stress index; PBMC—peripheral blood mononuclear cells; PMNs—polymorphonuclear cells; PN—parenteral nutrition; SMOF—soybean oil/MCT/olive oil/fish oil; SO—soybean oil; TAC—total antioxidant capacity; TAS—total antioxidant status; TBARS—thiobarbituric acid reactive substances; TOS—total oxidant status; TRAP—total radical-trapping antioxidant potential.

**Table 4:** Effects of olive oil-based intravenous lipid emulsions on plasma cholesterol and triglyceride levels.

Study	Population	Intervention and Control (*n*) (Lipid Dose)	Duration	Outcomes
**Animal studies**				
Harvey et al., 2014 [5]	Guinea pigs	OO SO FO SMOF	6 h infusion 10 d infusion	During 6 h infusion, TG increased significantly in all groups; however, greatest increase in SO group During chronic administration (10 d), TG was significantly lower in SO, OO, and FO lipids compared with SMOF and control diet groups (*p* < 0.05)
**Adult studies**				
García-de-Lorenzo et al., 2005 [35]	Severe burns	OO (*n* = 11) (1.3 g/kg/day) MCT/LCT (*n* = 11) (1.3 g/kg/day)	6 day	TG increased from baseline significantly in both groups and TC increased from baseline significantly in OO group. Between-group differences were not significant TC levels remained within normal ranges * for most patients
Gultekin et al., 2014 [69]	Sepsis or septic shock	OO (*n* = 16) (1.3 g/kg/day) OO + FO {90%/10%} (*n* = 16) (1.3 g/kg/day)	5 day	No difference from baseline to final measurement for TC, TG, LDL, or VLDL in both groups. HDL significantly decreased from baseline to final measurement in the OO group (*p* < 0.05) In OO group, TC and TG were within normal ranges * at baseline and final measurement
Huschak et al., 2005 [70]	Trauma	OO (*n* = 18) (0.8 g/kg/day) SO (*n* = 15) (0.5 g/kg/day)	14 day	No difference between groups in TG A significant difference in lipid dose delivered was observed between the groups (*p* < 0.001)
Olthof et al., 2013 [36]	Long-term PN	OO (*n* = 20) (NR) Healthy controls (*n* = 21)	≥6 months	TG levels were significantly higher in the PN group; however, TG levels were within normal ranges (as specified in the article) for both groups
Onar et al., 2011 [38]	Abdominal surgery (oncology)	OO (*n* = 10) (0.75 g/kg/day) SO (*n* = 10) (0.75 g/kg/day)	7 day	TC, LDL, VLDL, and HDL decreased from baseline in the OO group, no significant difference between groups All values were within normal ranges *
Pálová et al., 2008 [73]	Malnourished ≥10% decreased bodyweight	OO (*n* = 11) (NR) SO (*n* = 10) (NR)	14 day	TG deteriorated^†^ in 1/11 patients in OO group vs. 7/10 in SO group (*p* < 0.01)
Piper et al., 2009 [74]	Abdominal or major maxillofacial surgery (oncology)	OO (*n* = 22) (NR) SMOF (*n* = 22) (NR)	5 day	TG increased from baseline in both groups, and the increase was greater in the OO group. TG levels remained mostly within normal range * Significant between-group differences at Day 2 (*p* < 0.03) and Day 5 (*p* < 0.01)
Puiggròs et al., 2009 [71]	Abdominal surgery	OO (*n* = 7) (1.1–1.2 g/kg/day) SO (*n* = 7) (1.1–1.2 g/kg/day) MCT/LCT {50%/50%} (*n* = 7) (1.1–1.2 g/kg/day) MCT/LCT {36%/64%} (*n* = 7) (1.1–1.2 g/kg/day)	5 day	No change from baseline in TC, HDL, LDL, or TG in OO group, all values within normal ranges. No difference between groups for any of these measures
Reimund et al., 2005 [39]	Long-term PN	OO (*n* = 14) (31% of calories)	3 months	No change from baseline in TC, HDL, LDL, or TG in OO group No difference between groups for any of these measures Baseline and 3-month values within normal ranges for all measures
Siqueira et al., 2011 [24]	Healthy volunteers	OO (NR) SO (NR) Lipid-free PN Saline	24-h infusion of each intervention (random order) ^‡^	TG significantly increased from baseline in OO and SO groups compared with saline No difference between OO and SO groups in TC, HDL, or LDL between groups All values were within normal range *
**Pediatric studies**				
Goulet et al., 1999 [51]	Long-term PN	OO (*n* = 9) (1.92 g/kg/day) SO (*n* = 9) (1.69 g/kg/day)	Mean >30 months	TC, HDL, LDL, and TG decreased in OO group and increased in SO group Differences between groups not significant except for TC and LDL
Hartman et al., 2009 [62]	Bone marrow transplant	OO (*n* = 15) (1.1 g/kg/day) MCT/LCT (*n* = 13) (1.1 g/kg/day)	14 day	TC decreased from baseline in both groups; however, the decrease was significantly greater in the OO group (*p* = 0.017). TG decreased from baseline in both groups, but the difference between groups was not significant TC remained within normal ranges in both groups, TG was above normal range * at baseline and decreased to within normal range in the OO group. TG remained within normal ranges in MCT/LCT group
Kurvinen et al., 2011 [75]	Intestinal failure	OO (*n* = 11) (0.9 g/kg/day) Normal controls (*n* = 20)	3 months	TC was significantly lower in the OO group; however, TC remained within normal range * in both groups
**Preterm neonate studies**				
Demirel et al., 2012 [41]	≤32 week	OO (*n* = 20) (up to 3 g/kg/day) SO (*n* = 20) (up to 3 g/kg/day)	14 day	TC and TG within normal ranges * in both groups. No significant differences between groups except for VLDL, which was significantly higher in the OO group (*p* < 0.05), baseline levels NR
Koksal et al., 2011 [43]	≤34 week	OO (*n* = 32) (up to 3 g/kg/day) SO (*n* = 32) (up to 3 g/kg/day)	7 day	TC, VLDL, and TG increased, no significant difference between groups. All measures were within normal ranges *
Pitkanen et al., 2004 [63]	28–33 week	OO (0.48 g/kg/day) MCT/LCT (0.48 g/kg/day)	3 h ^§^	TG increased significantly (*p* < 0.001) in both groups
Wang et al., 2016 [46]	<2000 g and <37 week	OO (*n* = 50) (1.42 g/kg/day) MCT/LCT (*n* = 50) (1.30 g/kg/day) SO (*n* = 50) (1.39 g/kg/day)	>14 day	No significant differences were observed in TC, TG, apolipoprotein A-I, apolipoprotein B, Lp(a), and apolipoprotein A-I/B among the groups. However, on Day 7, HDL level in the MCT/LCT group (0.89 ± 0.31 mmol/L) was significantly lower than in the OO (1.06 ± 0.40 mmol/L) or SO (1.05 ± 0.33 mmol/L) groups (*p* < 0.05). On Day 7, LDL levels were significantly higher in OO (1.77 ± 0.44 mmol/L) than in MCT/LCT (1.58 ± 0.44 mmol/L) or SO (1.54 ± 0.38 mmol/L) groups (*p* < 0.05). TC, TG, HDL, and LDL levels were within normal ranges *

* Normal ranges based on ATPIII values: TG <150 mg/dL (1.69 mmol/L); TC 150–199 mg/dL (3.88–5.15 mmol/L); HDL ≥40 mg/dL (1.04 mmol/L); LDL ≤130 mg/dL (3.36 mmol/L) [68]. ^†^ Deterioration defined as a patient who moved to a more abnormal category after starting PN. Categories: within normal limits, elevation up to 2 × ULN, and elevation > 2 × ULN. ^‡^ Patients (*n* = 12) received a 24-h infusion of each lipid emulsion (in random order) on 2 consecutive days. ^§^ Patients (*n* = 13) received a 3-h infusion of each lipid emulsion (in random order) on 2 consecutive days. ATPIII—Adult Treatment Panel III; HDL—high-density lipoprotein; LDL—low-density lipoprotein; MCT/LCT—medium-chain triglycerides/long-chain triglycerides; NR—not reported; OO—olive oil; SMOF—soybean oil/MCT/olive oil/fish oil; SO—soybean oil; TC—total cholesterol; TG—triglycerides; ULN—upper limit of normal range; VLDL—very low-density lipoprotein.

**Table 5:** Effects of olive oil-based intravenous lipid emulsions on markers of liver function.

Study	Population	Intervention and Control (*n*) (Lipid Dose)	Duration	Outcomes
**Adult studies**				
Badía-Tahull et al., 2010 [33]	Gastrointestinal surgery	OO (*n* = 14) (0.88 g/kg/day) OO + FO {84% + 17%} (*n* = 13) (0.88 g/kg/day)	5 day	No significant differences between groups in LFTs (ALT, ALP, and GGT) at Day 6
García-de-Lorenzo et al., 2005 [35]	Severe burns	OO (*n* = 11) (1.3 g/kg/day) MCT/LCT (*n* = 11) (1.3 g/kg/day)	6 day	At Day 6, of 11 MCT/LCT and 9 OO patients, more in the MCT/LCT group had abnormal LFTs: ALT 8 vs. 4, AST 6 vs. 5, ALP 7 vs. 3, GGT 9 vs. 6, and bilirubin (total or conjugated) 4 vs. 2 Markers of cholestasis * in a significantly greater proportion of MCT/LCT vs. OO group (9/11 vs. 3/9, *p* = 0.04, Suissa-Shuster test) Markers of cytolysis^†^ associated with cholestasis in 3 MCT/LCT and 2 OO patients
Grau et al., 2007 [88]	ICU	OO or MCT/LCT (*n* = 303 initial TPN group) (NR) EN (*n* = 422) (NR)	Not pre-specified	Multivariate analysis showed TPN is significantly associated with LD (*p* < 0.001) LD in 91/303 (30%) TPN patients LD in 75/233 (32%) TPN patients receiving MCT/LCT Multivariate model found no relationship between ILE used and liver dysfunction Median duration of TPN was 5 d for patients with LD vs. 0 d for those without LD (*p* = 0.001) Cholestasis occurred in 31/303 (10%) TPN patients
Jia et al., 2015 [20]	ICU	OO (*n* = 226) (0.8 g/kg/day) SO (*n* = 232) (0.8 g/kg/day)	5–14 day	LFTs generally within normal limits ALT change from BL significantly greater in OO vs. SO group at Day 5 (*p* = 0.002) and EOT/Day 14 (*p* = 0.006) ALP and GGT changes from BL significantly greater in OO vs. SO group at Day 5 (*p* = 0.001 and *p* = 0.004, respectively), but not at EOT/Day 14. Increases in both enzymes suggest OO and SO associated with mild cholestasis No significant differences between groups for AST and total bilirubin Short-term PN with OO or SO did not appear to negatively impact liver function
Klek et al., 2017 [93]	Long-term PN	OO (*n* = 17) (0.6 g/kg/day) SO (*n* = 14) (0.7 g/kg/day) MCT/LCT {50% + 50%} (*n* = 18) (0.7 g/kg/day) SMOF (*n* = 16) (0.7 g/kg/day)	12 months	No significant change from BL for ALT, AST, AP, or GGT for SO, MCT, or SMOF. Bilirubin and GGT significantly decreased from BL (*p* = 0.0023 and *p* = 0.0079) in OO group; ALT, AST, and AP remained unchanged
Onar et al., 2011 [38]	Abdominal surgery (oncologic)	OO (*n* = 10) (0.75 g/kg/day) SO (*n* = 10) (0.75 g/kg/day)	7 day	ALP and GGT significantly increased from BL (*p* < 0.05) in both groups at Day 7 Total bilirubin significantly decreased from BL (*p* < 0.05) in OO group at Day 7 No significant change from BL for ALT and AST in both groups nor for total bilirubin in SO group at Day 7 No significant differences between groups for LFTs Increases in LFTs (AST, ALT, ALP, and bilirubin) occurred in 10% of patients; abnormalities resolved post PN
Pálová et al., 2008 [73]	Malnourished ≥10% decreased body weight	OO (*n* = 11) (NR) SO (*n* = 10) (NR)	14 day	No significant difference between groups in number of patients with deterioration ^‡^ in cytosolic enzymes (1 SO [ALT] vs. 1 OO [AST]) Significantly more patients with deterioration ^‡^ in cholestatic enzymes in SO vs. OO group (5 vs. 1, *p* < 0.05): conjugated bilirubin 3 vs. 0, ALP 3 vs. 1, and GGT 6 vs. 1
Piper et al., 2009 [74]	Abdominal surgery or major maxillofacial surgery	OO (*n* = 22) (NR) SMOF (*n* = 22) (NR)	5 day	Mean AST significantly lower in SMOF vs. OO group at Day 2 (27 vs. 47 U/L, *p* < 0.02) and Day 5 (31 vs. 56 U/L, *p* < 0.02) Mean ALT significantly lower in SMOF vs. OO group at Day 2 (20 vs. 42 U/L, *p* < 0.03) and Day 5 (26 vs. 49 U/L, *p* < 0.03) Mean α-GST significantly lower in SMOF vs. OO group at Day 2 (5 vs. 17 µg/L, *p* < 0.03) and Day 5 (6 vs. 24 µg/L, *p* < 0.01)
Puiggròs et al., 2009 [71]	Abdominal surgery	OO (*n* = 7) (1.1–1.2 g/kg/day) SO (*n* = 7) (1.1–1.2 g/kg/day) MCT/LCT {50%/50%}) (*n* = 7) (1.1–1.2 g/kg/day) MCT/LCT {37%/63%} (*n* = 7) (1.1–1.2 g/kg/day)	5 day	No significant differences between groups in changes from BL to Day 6 for LFTs (ALT, AST, ALP, GGT, and total bilirubin) A tendency (NS) to increase GGT in the SO and MCT/LCT structured groups and AST in the MCT/LCT mixture group at Day 6 was observed; however, values remained within normal limits
Reimund et al., 2005 [39]	Long-term PN	OO (*n* = 14) (31% of calories)	3 months	No significant changes from BL in bilirubin (total and conjugated), AST, ALT, ALP, and GGT at Month 3
Thomas-Gibson et al., 2004 [90]	Long-term PN	OO (*n* = 13) (up to 1 g/kg/day)	6 months SO followed by 6 months OO followed by 6 months SO ^§^	In 12 patients with >2 mo OO PN, bilirubin was within normal limits and AST was ≤15% outside normal range at BL. LFTs increased transiently in 4 patients and were persistently high in 1 severely septic patient who also had abnormal levels at baseline 1 new case of cholelithiasis was identified No biliary outflow abnormality at BL or endpoint of OO PN despite 6 patients having BL biliary disease In 11 patients with >2 mo SO PN after OO PN, no significant changes in LFTs occurred in 6 mo post OO PN
Vahedi et al., 2005 [91]	Long-term PN	MCT/LCT {50%/50%)}run-in, followed by OO (*n* = 6) (0.7 g/kg/day) SO (*n* = 7) (0.7 g/kg/day)	3 months	No differences between groups in changes in LFTs from BL to Day 90 1 case of cholestasis (SO) and 1 case of cytolysis (OO) existing at BL had resolved at Day 90 Hepatic ultrasound on Day 90 detected no hepatobiliary changes compared with BL
Wang et al., 2013 [92]	Resectable esophageal cancer	EN + OO PN (*n* = 46) (~0.83 g/kg/day) EN + MCT/LCT PN (*n* = 48) (~0.83 g/kg/day)	PN 7 day, EN added after Day 7	Liver function was measured at regular intervals; results not reported
**Pediatric studies**				
Goulet et al., 1999 [51]	Long-term PN	OO (*n* = 9) (1.92 g/kg/day) SO (*n* = 9) (1.69 g/kg/day)	Mean >30 months	No significant differences between groups in changes from BL to Day 60 in bilirubin (total and conjugated), LFTs (AST, ALT, ALP, and GGT) and biliary acids Total bilirubin increased from BL in both groups ALT increased from BL in both groups AST increased from BL in OO group and decreased in SO group ALP increased from BL in OO group and decreased in SO group GGT essentially unchanged in OO group and increased from BL in SO group Biliary acids increased from BL in OO group and essentially unchanged in SO group
Hartman et al., 2009 [62]	Bone marrow transplant	OO (*n* = 15) (1.1 g/kg/day) MCT/LCT (*n* = 13) (1.1 g/kg/day)	14 day	No significant differences for LFTs between groups
Kurvinen et al., 2011 [75]	Long-term PN	OO (*n* = 11) (0.9 g/kg/day)	>3 months	ALT, AST, GGT, and bilirubin remained close to normal or within the normal range during follow-up ^#^ GGT correlated with serum PS (*r* = 0.61–0.62, *p* < 0.05). Liver biopsies showed fibrosis in 5/8 (63%) patients and cholestasis in 3/8 (38%) patients Liver fibrosis in 5 patients reflected increased serum PS (*r* = 0.55–0.60, *p* = 0.16–0.12)
**Preterm neonate studies**				
Demirel et al., 2012 [41]	<32 wk	OO (*n* = 20) (up to 3 g/kg/day) SO (*n* = 20) (up to 3 g/kg/day)	14 day	LFTs normal and similar in both groups at 14th day of life
Deshpande et al., 2014 [64]	<30 wk	OO (*n* = 17) (18.45 g/kg/day) SMOF (*n* = 17) (18.25 g/kg/day)	7 day	No significant difference between groups in bilirubin (total and conjugated) or LFTs (ALT and GGT) on Day 8; values within normal limits in both groups
Gobel et al., 2003 [87]	NICU patients, gestational age 28–36 wk	OO (*n* = 18) (up to 2 g/kg/day) SO (*n* = 15) (up to 2 g/kg/day)	7 day	No significant differences between groups for changes from BL to Day 8 for LFTs (bilirubin [total and conjugated], AST, ALT, ALP, and GGT) AST significantly lower at Day 8 vs. BL in both groups (OO: 14.2 vs. 27.2 IU/L, *p* = 0.0001; SO: 13.9 vs. 25.4 IU/L, *p* = 0.0007) ALT lower at Day 8 vs. BL (NS) in both groups ALP significantly higher at Day 8 vs. BL in both groups (OO: 286 vs. 222 IU/L, *p* = 0.0039; SO: 269 vs. 207 IU/L, *p* = 0.0028) GGT significantly lower at Day 8 vs. BL in both groups (OO: 64.0 vs. 75.0 IU/L, *p* = 0.0139; SO: 63.8 vs. 83.3 IU/L, *p* = 0.0073) Total bilirubin lower at Day 8 vs. BL (NS) in both groups Conjugated bilirubin higher at Day 8 vs. BL in OO group (NS) and lower in SO group (NS)
Koksal et al., 2011 [43]	≤34 wk	OO (*n* = 32) (up to 3 g/kg/day) SO (*n* = 32) (up to 3 g/kg/day)	7 day	AST, ALT, and bilirubin (total and indirect) decreased from BL to Day 7 while ALP and GGT increased in both groups (NS) No significant differences between groups in LFTs
Savini et al., 2013 [44]	500–1249 g	OO (*n* = 29) (up to 3 g/kg/day) SO (*n* = 30) (up to 3 g/kg/day) MCT/LCT (*n* = 30) (up to 3 g/kg/day) MSF (*n* = 27) (up to 3 g/kg/day) SMOF (*n* = 28) (up to 3 g/kg/day)	21 day	No significant differences between groups in mean AST, ALT, ALP, GGT, or bilirubin (total and conjugated) at 6 weeks of age Cholestasis (conjugated bilirubin >2.0 mg/dL) in 3 (2.1%) patients (1 MCT/LCT, 1 MSF, 1 SMOF) at 6 weeks of age, when all infants were receiving minimal enteral feeding No significant correlations between phytosterol intake, conjugated bilirubin, and LFTs at 6 weeks of age
Wang et al., 2016 [45]	<2000 g and <37 wk	OO (*n* = 50) (1.45 g/kg/day) SO (*n* = 50) (1.41 g/kg/day)	>14 day	Mean total bilirubin elevated at BL in both groups (OO 2.75 mg/dL, SO 38.80 mg/dL). At Day 7, mean values significantly increased in OO group (8.35 mg/dL) and significantly decreased in SO group (9.00 mg/dL) (*p* < 0.05). At Day 14, mean values significantly decreased from Day 7 in both groups (OO 4.13 mg/dL, SO 3.83 mg/dL) (*p* < 0.05) Direct bilirubin elevated at BL in both groups (OO 0.55 mg/dL, SO 0.59 mg/dL) and significantly increased at Day 7 (1.01 mg/dL, *p* < 0.05) and Day 14 (0.67 mg/dL) in SO group; however, increases not significant in OO group (0.75 and 0.73 mg/dL) Direct bilirubin significantly different between groups (*p* = 0.039) ALT not significantly different from BL at Days 7 and 14 in both groups; mean values remained within normal limits At Days 7 and 14, AST significantly decreased into normal range from high values at BL (*p* < 0.05) in both groups. ALP similarly elevated at BL and similarly increased significantly at Days 7 and 14 in both groups (*p* < 0.05) Mean GGT elevated at BL in both groups (OO 98 IU/L, SO 215 IU/L). At Days 7 and 14, mean GGT significantly increased from BL in OO group (100 and 139 IU/L) and significantly decreased in SO group (112 and 89 IU/L) (*p* < 0.05) No significant differences between groups for ALT, AST, ALP, GGT, and total bilirubin
Wang et al., 2016 [46]	<2000 g and <37 wks	OO (*n* = 50) (1.42 g/kg/day) MCT/LCT (*n* = 50) (1.30 g/kg/day) SO (*n* = 50) (1.39 g/kg/day)	>14 day	Total and direct bilirubin highest at Day 7 in all groups AST decreased from high values at BL to within normal limits in all groups at Days 7 and 14 ALT remained within normal limits in all groups ALP elevated at BL and increased at Days 7 and 14 in all groups GGT elevated at BL and decreased but remained elevated at Days 7 and 14 in all groups No significant differences in LFTs among groups at BL and Days 7 and 14
Webb et al., 2008 [61]	≥25 wk	OO (*n* = 39) (23.1 kcal/kg/day) SO (*n* = 39) (24.3 kcal/kg/day)	5 day	LFTs were similar in both groups at BL and Day 5 No abnormalities or differences between groups in ALP, GGT, or conjugated bilirubin at BL or Day 5 Bile acids increased at Day 5 in both groups; no difference between groups
**Systematic literature reviews and meta-analyses**				
Dai et al., 2016 [83]	SLR and meta-analysis of RCTs: Neonates, infants, children, and adults	OO 8 studies SMOF 7 studies SO (control in each study)	Various	No differences for any analyses of total bilirubin. ALP significantly higher in OO vs. SO group (infants plus children, *p* < 0.00001) AST and ALP significantly lower in SMOF vs. SO group (all ages combined, *p* = 0.004 and *p* = 0.02) ALT, AST, and ALP significantly lower in SMOF vs. SO group (only adults, *p* = 0.004, *p* = 0.006, and *p* = 0.03) GGT lower in SMOF vs. SO group (all ages combined and only adults, *p* = 0.07 and *p* = 0.08)
Edward et al., 2017 [66]	SLR of 17 RCTs in hospitalized pediatric patients	OO 7 studies (control in 1 study control) SMOF 8 studies SO (control 15 studies) MCT/LCT 2 studies (control in 1 study) FO 2 studies	Various	The evidence does not point toward a particular ILE being superior in terms of effect on liver enzymes or total bilirubin. The majority of studies did not find significant differences between use of different ILEs and liver enzymes
Hojsak et al., 2016 [81]	SLR and meta-analysis of 23 RCTs: Preterm neonates, infants, and children	OO 2 studies SMOF 4 studies SMF 1 study MCT 1 study SO (control)	Various	Meta-analysis showed no differences in the rate of cholestasis or bilirubin levels associated with short-term use of different ILEs in preterm infants, neonates, and children Some evidence that use of multicomponent FO-containing ILE may contribute to a decrease in total bilirubin levels in children with intestinal failure on long-term PN

* Cholestasis defined as a value > ULN for 2 of 3 parameters (conjugated bilirubin, AP, and GGT) during treatment. ^†^ Cytolysis defined as a value > 2 × ULN for AST and/or ALT during treatment. ^‡^ Deterioration defined as a patient who moved to a more abnormal category after starting PN. Categories: within normal limits, elevation up to 2 × ULN, and elevation > 2 × ULN. ^§^ *n* = 12. ^#^ Excluded 1 patient with >20-fold increase in serum PS decreased together with ALT and bilirubin after transition from SO-based to OO-based PN after inclusion in the study. α-GST—alpha-glutathione *S*-transferase; ALP—alkaline phosphatase; ALT—alanine aminotransferase; AST—aspartate aminotransferase; BL—baseline; EN—enteral nutrition; EOT—end of treatment; FO—fish oil; GI—gastrointestinal; GGT—gamma-glutamyl transpeptidase; ILE—intravenous lipid emulsion; LCT—long-chain triacylglycerol; LD—liver dysfunction; LFT—liver function test; MCT—medium-chain triacylglycerol; MSF—50% MCTs/40% SO/10% FO; NEC—necrotizing enterocolitis; NICU—neonatal intensive care unit; OO—olive oil; OR—odds ratio; PN—parenteral nutrition; postop—postoperatively; PNALD—PN-associated liver disease; PS—phytosterol; RCT—randomized controlled trial; SBS—short-bowel syndrome; SLR—systematic literature review; SMOF—30% SO/30% MCT/25% OO/15% FO; SO—soybean oil; TG—triglyceride; ULN—upper limit of normal range; VLBW—very low birth weight.
